# Behavioral Trait of Morningness-Eveningness in Association with Articular and Spinal Diseases in a Population

**DOI:** 10.1371/journal.pone.0114635

**Published:** 2014-12-03

**Authors:** Ilona Merikanto, Tuuli Lahti, Seppo Seitsalo, Erkki Kronholm, Tiina Laatikainen, Markku Peltonen, Erkki Vartiainen, Timo Partonen

**Affiliations:** 1 Department of Mental Health and Substance Abuse Services, National Institute for Health and Welfare, Helsinki, Finland; 2 Department of Biosciences, University of Helsinki, Helsinki, Finland; 3 Orton Orthopaedics Hospital, Helsinki, Finland; 4 Department of Behavioural Sciences and Philosophy, University of Turku, Turku, Finland; 5 Department of Chronic Disease Prevention, National Institute for Health and Welfare, Helsinki and Turku, Finland; 6 Institute of Public Health and Clinical Nutrition, University of Eastern Finland, Kuopio, Finland; 7 Hospital District of North Karelia, Joensuu, Finland; San Francisco Coordinating Center, United States of America

## Abstract

Earlier studies have revealed that the more the preference to schedule daily activities towards the evening hours is, the higher the odds for a range of health hazards are. Therefore, we wanted to analyze, whether the behavioral trait of morningness-eveningness is associated with articular and spinal diseases or those with musculoskeletal disorders. Participants (n = 6089), as part of the National FINRISK 2007 Study, were derived from the general population, aged 25 to 74 years, living in Finland. Chronotype was assessed based on six items from the original Horne-Östberg Morningness-Eveningness Questionnaire. Information about risk factors and the diagnoses of articular and spinal diseases were based on the self-reported information. Our results suggest that Evening-types have higher odds for articular and spinal diseases as compared with Morning-types, and this risk is heightened especially regarding spinal disease and backache (odds ratios of 1.8 to 2.1, and 1.6 to 1.8, respectively) and remains significant after controlling for the sex, age, education, civil status, physical activity, alcohol use, and smoking, and additionally for the body-mass index, insufficient sleep, or depressive symptoms.

## Introduction

Individuals display their preference for timing of the daily activities early in the morning (morningness) in the one end and late in the evening (eveningness) in the other end. This behavioral trait is a continuum, and based on the position taken on the scale, humans can be classified as the earlier-timed Morning-types (M-types), the later-timed Evening-types (E-types), or the Intermediate-types (I-types) whose preference for timing of the daily activities is intermediate between the two extremes [1]-[3]. Both intrinsic and environmental factors contribute to this behavioral trait (or chronotype), reflecting interaction between the intrinsic circadian clocks and the external social clock. The estimate for overall genetic effect (broad sense heritability) was 49.7% in a nationwide twin study, leaving environmental factors not shared by siblings to account for the remainder [4].

Previous studies have revealed that E-types have higher risks for a range of health problems [5]–[10], they are more prone to worse health habits such as smoking [11]–[14], greater alcohol use [11] and binge drinking [15], and dietary intakes that are not only unhealthy [16] but also emotionally-driven [17]. Further, E-types also tend to die younger [18], but concerning this issue, however, there are currently only limited data (i.e. one prospective cohort dataset) on men only.

Sleep disturbances and depression are common in patients with rheumatoid arthritis and those with osteoarthritis [19]–[21]. Population-based prospective cohort studies have identified the body-mass index (BMI) and physical activity at work as major risk factors of primary osteoarthritis, and of subsequent knee replacement, these two risk factors being additive [22], and after adjustment for diffuse osteoarthritis, non-restorative sleep is the strongest predictor of newly-emerged widespread pain [23]. By identifying the broad range of risk factors and quantifying them in a population, it becomes feasible to take steps to prevent diseases more effectively [24]. In rheumatoid arthritis, rheumatic symptoms, and osteoarthritis there are diurnal variation in symptom severity, suggesting that the intrinsic circadian clock and thus the chronotype might influence the pathogenesis [25]–[27]. Moreover, as earlier studies have thus far demonstrated that E-types have insufficient and fragmented sleep more frequently as well as a higher risk of depression than others [7], [9], there is a rationale to study, whether the chronotype contribute to rheumatoid arthritis and osteoarthritis.

### Aims

In this study, considering the higher risks observed among E-types for sleeping problems and depression that are common among patients having rheumatoid arthritis or osteoarthritis, we wanted to analyze whether eveningness is associated with a heightened risk for articular and spinal diseases. To meet these research questions, our sample was a big one and derived from the general population, aged 25 to 74 years, living in Finland.

## Methods

### Participants

A random sample of 10,000 inhabitants, aged 25 to 74 years, was invited to the National FINRISK Study 2007 from five large geographical areas in Finland. The target sample was stratified according to the gender, the 10-year age groups and the geographical areas based on the information provided by the Finnish Population Information System. From each area 2000 participants were asked to fill in the study questionnaires, which they received beforehand by mail, and to participate in a health examination organized in a local health care center from 22 January 2007 to 30 March 2007. We present here data from participants with the complete information on chronotype available (n = 6089).

### Assessment

Chronotype was assessed in the National FINRISK Study 2007 questionnaire with six questions (MEQ-items 4, 7, 9, 15, 17 and 19) derived from the original 19-item Morningness-Eveningness questionnaire (MEQ) that correlate with the intrinsic period of the circadian pacemaker [1]. In regression analyses, these six items explain 83% of the variation in the original MEQ sum score, their internal consistency being Cronbach's alpha of 0.80 [28]. The sum score of Morningness-Eveningness was divided in three categories, consisting of definitely or moderately Evening-types (5 to 12), Intermediate-type (13 to 18), and definitely or moderately Morning-types (19 to 27). This scoring corresponds the original MEQ scaling, where higher sum scores correlate with tendency for morningness while lower sum scores correlate with tendency for eveningness [2], [3].

In the questionnaire, sufficient sleep was asked as follows: “Do you think you sleep enough?” (Yes, nearly always; Yes, often; Rarely; I cannot say). Depressive symptoms were assessed by combining the following two questions into three dichotomized categories: none vs. one or two symptoms: 1. “Have you had during the last 12 months at least a two-week continuous period when you have felt dispirited or depressed?” (Yes, No); 2. “Have you had during the last 12 months at least a two-week continuous period when you have lost interest in most of the things that normally feel good, such as hobbies or work?” (Yes, No).

Data for risk factors and the diagnoses of articular and spinal diseases comprised of the self-reported information from the questionnaires as follows: 1. “In the past 12 months, has doctor diagnosed or treated you for rheumatoid arthritis?” (Yes, No); 2. “In the past 12 months, has doctor diagnosed or treated you for other articular disease?” (Yes, No); 3. “In the past 12 months, has doctor diagnosed or treated you for spinal fractures or other spinal diseases?” (Yes, No); 4. “In the past month, have you had rheumatic symptoms?” (Yes, No); 5. “In the past month, have you had articular pain?” (Yes, No); 6. “In the past month, have you had backache?” (Yes, No); 7. “When was the last time you have used medication for articular pain?” (Past week; 1–4 weeks ago; 1–12 months ago; Over a year ago; Never).

### Data analysis

Two-sided chi-square tests and t-tests, as appropriate, were used to evaluate the difference in key sociodemographic, socioeconomic and health characteristics of chronotypes (Table 1).

**Table 1:** Sociodemographic, socioeconomic and health characteristics by chronotype.^a^

	Chronotype, N = 6089		
	Evening, N = 730	Intermediate, N = 2507	Morning, N = 2852
**Gender** (%)****			
Men	41.6	43.1	49.8
Women	58.4	56.9	50.2
			
**Age** (years, mean ± s.d)****	43.8±13.4	48.1±14.0	53.1±13.0
**Education level** (%)****			
Basic	14.5	18.4	30.4
Secondary	55.5	55.0	53.0
Higher	30.0	26.6	16.6
**Civil status** (%)****			
Married	41.1	54.6	60.4
Co-habiting	20.0	18.0	14.0
Unmarried	25.8	14.6	11.4
Separated	11.5	9.6	10.0
Widow	1.6	3.2	4.2
**Physical activity** (%)****			
Never	3.9	3.9	3.2
Less than once a week	25.0	16.5	14.1
Once a week	16.4	15.6	13.6
Two times a week	20.3	20.8	17.9
Three times a week	18.2	20.8	20.6
Four times a week	8.3	11.9	14.8
Five times a week or more	8.0	10.6	15.8
**Smoking** (%)****			
Never regularly or stopped over ½ years ago	64.8	77.4	79.3
Smokes or stopped less than ½ years ago	35.2	22.6	20.7
**Smoking** (%)****			
Never regularly	47.2	56.5	53.3
Smokes presently or has smoked regularly	52.8	43.5	46.7
**Alcohol consumption** (%)****			
At least once a month	71.2	68.5	64.2
Less than once a month	19.3	21.5	22.6
Not any more	5.5	4.0	5.4
Never used alcohol	4.0	6.0	7.8
**BMI** (mean ± s.d)****	26.8±5.3	26.6±4.8	27.4±4.8
Do you think you sleep enough?****			
Yes, nearly always	19.1	29.6	46.1
Yes, often	38.3	47.2	40.7
Rarely	32.2	16.2	7.7
I cannot say	10.4	6.9	5.5
Depressive symptoms****			
No depressive symptoms	57.1	74.4	83.7
One depressive symptom	16.0	10.9	7.1
Two depressive symptoms	26.9	14.7	9.2

a Abbreviation: s.d.  =  standard deviation. Chi-square tests for non-parametric data, and t-test for parametric data, where ^*^ *p*<0.05; ^**^ *p*<0.01; ^***^ *p*<0.001; ^****^ *p*<0.0001.

Logistic regression analyses were used to estimate the odds ratios (ORs) with 95% confidence limits (CLs) for the self-reported spinal diseases and medication by chronotype, using Morning-types as the reference category. The information regarding medication for articular pain was changed in the analysis to a binary item (a person has ever used medication or has never used medication for articular pain).

First, the crude (univariate) association with chronotype was analyzed (model 1); second, the model was controlled for gender and age (model 2); and finally, for gender, age, education level, civil status, physical activity, alcohol consumption, and current smoking (model 3 whose covariates are described in more detail in Table 1).

In supplementary analysis, the BMI (Table S1), depressive symptoms (Table S2) or insufficient sleep (Table S3) was included as the explanatory variable in the final model (model 3).

### Ethics

The plan of the National FINRISK Study 2007 was approved by the Ethics Committee (Institutional Review Board) of the Hospital District of Helsinki and Uusimaa, Finland (#20.2.2007/229/E0/06). The study was conducted according to accepted international ethical standards in accordance to the Declaration of Helsinki and its amendments. All the participants gave a written informed consent.

## Results

### Association of chronotype with articular diseases, symptoms and medication

E-types complained more rheumatic symptoms, articular pain and self-reported more other articular diseases diagnosed or treated by doctor in the past 12 months than M-types when controlled for gender and age (model 2) and multiple explanatory variables (model 3) (Table 2). In full model (model 3), E-types also reported more usage of medication for articular pain as compared with M-types.

**Table 2:** Articular diseases, symptoms and medication predicted by chronotype.^a^

Chronotype	Odds ratio	95% confidence limit	
		Lower	Upper
**Rheumatoid arthritis diagnosed or treated by doctor in the past 12 months**			
Model 1 (N = 6074, No N = 5988, Yes N = 86)			
Evening-types	0.6	0.2	1.5
Intermediate-types	1.7	1.1	2.6*
Model 2 (N = 6074, No N = 5988, Yes N = 86)			
Evening-types	0.9	0.3	2.2
Intermediate-types	2.0	1.3	3.1**
Model 3 (N = 5954, No N = 5873, Yes N = 81)			
Evening-types	0.9	0.3	2.4
Intermediate-types	2.0	1.3	3.3**
**Rheumatic symptoms past month**			
Model 1(N = 6079, No N = 5632, Yes N = 447)			
Evening-types	1.2	0.9	1.6
Intermediate-types	1.1	0.9	1.3
Model 2 (N = 6079, No N = 5632, Yes N = 447)			
Evening-types	1.7	1.2	2.2**
Intermediate-types	1.2	1.0	1.5
Model 3 (N = 5961, No N = 5527, Yes N = 434)			
Evening-types	1.6	1.1	2.2**
Intermediate-types	1.2	0.9	1.5
**Articular pain** **past month**			
Model 1(N = 6074, No N = 4167, Yes N = 1907)			
Evening-types	0.9	0.8	1.1
Intermediate-types	0.9	0.8	1.0*
Model 2 (N = 6074, No N = 4167, Yes N = 1907)			
Evening-types	1.3	1.1	1.5*
Intermediate-types	1.0	0.9	1.2
Model 3 (N = 5956, No N = 4093, Yes N = 1863)			
Evening-types	1.3	2.0	1.5*
Intermediate-types	1.0	0.9	1.2
**Other articular disease** **diagnosed or treated by doctor in the past 12 months**			
Model 1 (N = 6063, No N = 5444, Yes N = 619)			
Evening-types	0.9	0.7	1.1
Intermediate-types	0.8	0.7	1.0*
Model 2 (N = 6063, No N = 5444 Yes N = 619)			
Evening-types	1.3	1.0	1.8*
Intermediate-types	1.0	0.8	1.2
Model 3 (N = 5944, No N = 5337, Yes N = 607)			
Evening-types	1.4	1.0	1.8*
Intermediate-types	1.0	0.8	1.2
**Medication for articular pain**			
Model 1 (N = 5967, No N = 1577, Yes N = 4390)			
Evening-types	1.1	0.9	1.4
Intermediate-types	1.0	0.9	1.2
Model 2 (N = 5967, No N = 1577, Yes N = 4390)			
Evening-types	1.2	1.0	1.5
Intermediate-types	1.1	0.9	1.2
Model 3 (N = 5858, No N = 1545, Yes N = 4313)			
Evening-types	1.3	1.0	1.5*
Intermediate-types	1.1	0.9	1.2

a Model 1 crude (univariate); model 2 controlled for gender and age; model 3 controlled for gender, age, education level, civil status, physical activity, alcohol consumption, and current smoking. Morning-types as the reference category. ^*^ *p*<0.05; ^**^ *p*<0.01; ^***^ *p*<0.001; ^****^ *p*<0.0001.

When the influence of BMI was controlled for, no differences between E-types and M-types were seen regarding rheumatic symptoms and usage of medication for articular pain (Table S1).

When the influence of depression (Table S2) or insufficient sleep (Table S3) were controlled for, no differences between E-types and M-types were seen regarding rheumatic symptoms and usage of medication for articular pain as well as regarding articular pain and other articular diseases. Therefore, sleep problems and depressive symptoms seen in E-types (Table 1) contributed to the higher occurrence of articular diseases and symptoms in E-types as compared with M-types.

As compared with M-types, the intermediate I-types had the increased odds for a diagnosed or treated rheumatoid arthritis in all the models and for articular pain in crude analyses.

### Association of chronotype with spinal diseases and symptoms

E-types self-reported more diagnosed spinal disease when controlled for gender and age (model 2) or several characteristics (model 3). Furthermore, E-types reported more backaches than M-types in all the models (Table 3).

**Table 3:** Spinal diseases and symptoms predicted by chronotype.^a^

Chronotype	Odds ratio	95% confidence limit	
		Lower	Upper
**Spinal disease diagnosed or treated by doctor in the past 12 months**			
Model 1(N = 6063, No N = 5133, Yes N = 930)			
Evening-types	1.1	0.9	1.4
Intermediate-types	0.9	0.8	1.1
Model 2 (N = 6063, No N = 5133, Yes N = 930)			
Evening-types	1.5	1.2	1.9***
Intermediate-types	1.1	0.9	1.2
Model 3 (N = 5945, No N = 5035, Yes N = 910)			
Evening-types	1.6	1.2	2.0***
Intermediate-types	1.1	0.9	1.3
**Backache past month**			
Model 1(N = 6070, No N = 3417, Yes N = 2653)			
Evening-types	1.5	1.3	1.8***
Intermediate-types	1.2	1.1	1.3****
Model 2 (N = 6070, No N = 3417, Yes N = 2653)			
Evening-types	1.5	1.3	1.8****
Intermediate-types	1.2	1.1	1.4***
Model 3 (N = 5951, No N = 3351, Yes N = 2600)			
Evening-types	1.5	1.3	1.8***
Intermediate-types	1.2	1.1	1.4****

a Model 1 crude (univariate); model 2 controlled for gender and age; model 3 controlled for gender, age, education level, civil status, physical activity, alcohol consumption, and current smoking. Morning-types as the reference category. ^*^ *p*<0.05; ^**^ *p*<0.01; ^***^ *p*<0.001; ^****^ *p*<0.0001.

These results remained significant even after controlling for BMI, depressive symptoms or insufficient sleep. The intermediate I-types reported also more backaches than M-types (Tables S1–S3).

## Discussion

Our results are, to our knowledge, the first to report the association of chronotype with articular and spinal diseases and musculoskeletal disorders. Our study herein shows that eveningness is associated with greater odds for some of these diseases, such as spinal disease, articular diseases (other than rheumatoid arthritis), rheumatic symptoms, articular pain, and backache.

Stronger tendency to depression and sleep problems in E-types [7], [9] contribute according to our results to the greater occurrence of rheumatic symptoms, articular pain and higher usage of medication for articular pain in E-types. Further, they appear to account the higher risk for articular disease (other than rheumatoid arthritis) and bone and cartilage diseases. Population-based prospective studies have identified the BMI and physical activity (at work) as major risk factors of primary osteoarthritis [22], and after adjustment for diffuse osteoarthritis, non-restorative sleep as the strongest predictor of newly-emerged widespread pain [23]. Yet, after controlling for insufficient sleep and depressive symptoms, we found that E-types still had the increased odds for spinal disease and backache. This finding demonstrates that tendency towards eveningness is a high-risk factor regardless of tendency to sleep problems and depression for spinal disease.

Hypotheses of how sleep deprivation or depression results in an earlier onset of articular disorders due to lowered immune responses as well as increases in pain have been presented, but further mechanistic studies are needed to test and verify (or falsify) these hypotheses [19], [29]. Both sleep deprivation and depression have been related to low-grade inflammation and elevated cytokine levels in several studies [30]–[31]. In one study, sleep deprivation increased pain in patients with rheumatoid arthritis as compared to healthy subjects [20], and treatment of insomnia alleviated rheumatic symptoms in another study [19]. Some studies have found similar relationships between depression and pain in patients with rheumatoid arthritis, but a mechanism of action through which depression influences pain experience in these patients remain unclear [29].

Intriguingly, an overarching intrinsic factor might be a disturbance in circadian clock functions. In humans, on the one hand, synovial fibroblasts display alterations in several circadian clock components and in subsequent production of proinflammatory cytokines in rheumatoid arthritis [32]. On the other hand, in mice, genetic ablation of the key clock gene *Arntl* (or *Bmal1*) in a targeted tissue (the non-ciliated epithelial cells lining the bronchioles) leads to exaggerated innate inflammatory responses as well as impaired host responses to infection despite normal corticosteroid secretion [33]. If this kind of a mechanism were to hold for articular conditions as well, it might explain the link seen between chronotype and articular and spinal diseases.

Because the tendency towards eveningness is more common in younger persons [7], [34]–[35], it is not surprising that many results were not significant in the crude analyses. It is probable that younger people, belonging to a random sample of the general population aged 25 to 74 we analyzed herein, suffer less from articular diseases than older people.

One is left with the question, how our findings apply clinically? First, screening patients with the easy assessment of chronotype might help in early identification of not only poor sleep and depressive symptoms, but also in early detection of the potential risks for articular and spinal diseases. Second, whether E-types are more difficult to treat or whether M-types are more responsive to certain treatments, it remains to be tested.

All in all to conclude, eveningness seems to be associated with higher risk for spinal disease and articular diseases (other than rheumatoid arthritis). Among the E-types, the higher risk for articular diseases is to a great extent due to poor sleep and depressive symptoms. However, regarding spinal disease and backache, the increased odds are significant and self-contained, being independent of poor sleep and of depressive symptoms.

### Limitations and strengths

A limitation of our study is that the assessment of chronotype and information about the spinal and articular diseases and their symptoms was based on the self-report information only. There are more accurate assessment methods for chronotype, but the use of such methods would have been too challenging and expensive for a large sample of this study. With respect to the key outcomes variables, their causes remain unknown, their category remains undefined (herniated disc, lumbar stenosis, vertebral body fracture, etc.), and their chronicity and severity remain undefined. As the self-reported information about the diseases and their symptoms might not be reported accurately in all cases, it is clear that further studies based on objective measurements and diagnostic assessments are needed to clarify the association of spinal and articular diseases with eveningness. Such studies are also needed in order to elucidate the mechanisms by which eveningness and the evening chronotype associate with these diseases and the question whether the chronotype predisposes to these diseases.

On the other hand, the big population-based sample which is derived from a national health examination survey is a major strength of our study.

## Supporting Information

Table S1 **Supplementary analysis including BMI in the final model.**
(DOCX)

Table S2 **Supplementary analysis including depressive symptoms in the final model.**
(DOCX)

Table S3 **Supplementary analysis including insufficient sleep in the final model.**
(DOCX)
