# Supplementary material for: Behavioral Trait of Morningness-Eveningness in Association with Articular and Spinal Diseases in a Population
Source: PLoS One. 2014 Dec 3;9(12):e114635. doi: 10.1371/journal.pone.0114635 (PMC4255027; doi:10.1371/journal.pone.0114635)
Supplement: Table S1 — Supplementary analysis including BMI in the final model. (DOCX) [file pone.0114635.s001.docx]

Table S1. Supplementary analysis including BMI in the final model.^a^

| Chronotype | Odds ratio | 95% confidence limit | |
| --- | --- | --- | --- |
|  |  | Lower | Upper |
|  | | | |
| **Rheumatoid arthritis diagnosed or treated by doctor in the past 12 months** (N=5589, No N=5517, Yes N=72) | | | |
| Evening-types | 1.0 | 0.4 | 2.8 |
| Intermediate-types | 2.0 | 1.2 | 3.3** |
| **Rheumatic symptoms past month** (N=5593, No N=5195, Yes N=398) | | | |
| Evening-types | 1.6 | 1.2 | 2.2** |
| Intermediate-types | 1.2 | 0.9 | 1.5 |
| **Articular pain** **past month** (N=5589, No N=3829, Yes N=1760) | | | |
| Evening-types | 1.3 | 1.0 | 1.6* |
| Intermediate-types | 1.0 | 0.9 | 1.2 |
| **Other articular disease** **diagnosed or treated by doctor in the past 12 months** (N=5579, No N=5002, Yes N=577) | | | |
| Evening-types | 1.4 | 1.0 | 1.9* |
| \| Intermediate-types \|  \| \| \| --- \| --- \| --- \| \| Lower \| Upper \| \|  \|  \|  \| \|  \|  \|  \| \|  \|  \|  \| \|  \|  \|  \| \|  \|  \|  \| \|  \|  \|  \| \|  \|  \|  \| \|  \|  \|  \| \|  \|  \|  \| \|  \|  \|  \| \|  \|  \|  \| \|  \|  \|  \| \|  \|  \|  \| \|  \|  \|  \| \|  \|  \|  \| \|  \|  \|  \| \|  \|  \|  \| \|  \|  \|  \| \|  \|  \|  \| \|  \|  \|  \| \|  \|  \|  \| \|  \|  \|  \| \|  \|  \|  \| \|  \|  \|  \| \|  \|  \|  \| \|  \|  \|  \| \|  \|  \|  \| \|  \|  \|  \| \|  \|  \|  \| \|  \|  \|  \| \|  \|  \|  \| \|  \|  \|  \| \|  \|  \|  \| \|  \|  \|  \| \|  \|  \|  \| \|  \|  \|  \| \|  \|  \|  \| \|  \|  \|  \| \|  \|  \|  \| \|  \|  \|  \| \|  \|  \|  \| \|  \|  \|  \| \|  \|  \|  \| \|  \|  \|  \| \|  \|  \|  \| \|  \|  \|  \| \|  \|  \|  \| \|  \|  \|  \|   Intermediate-types | 1.0 | 0.8 | 1.2 |
| **Medication for articular pain** (N=5512, No N=1430, Yes N=4082) | | | |
| Evening-types | 1.2 | 1.0 | 1.5 |
| Intermediate-types | 1.1 | 0.9 | 1.2 |
| **Spinal disease diagnosed or treated by doctor in the past 12 months** (N=5579, No N=4726, Yes N=853) | | | |
| Evening-types | 1.6 | 1.3 | 2.1*** |
| Intermediate-types | 1.1 | 0.9 | 1.3 |
| **Backache past month** (N=5584, No N=3146, Yes N=2438) | | | |
| Evening-types | 1.5 | 1.2 | 1.8**** |
| Intermediate-types | 1.2 | 1.1 | 1.4** |

^a^ Controlled for gender, age, education level, civil status, physical activity, alcohol consumption, current smoking, and BMI. Morning-types as the reference category. ^*^*p* <0.05; ^**^*p* <0.01; ^***^*p* <0.001; ^****^*p* <0.0001
