# Supplementary material for: Behavioral Trait of Morningness-Eveningness in Association with Articular and Spinal Diseases in a Population
Source: PLoS One. 2014 Dec 3;9(12):e114635. doi: 10.1371/journal.pone.0114635 (PMC4255027; doi:10.1371/journal.pone.0114635)
Supplement: Table S2 — Supplementary analysis including depressive symptoms in the final model. (DOCX) [file pone.0114635.s002.docx]

Table S2. Supplementary analysis including depressive symptoms in the final model.^a^

| Chronotype | Odds ratio | 95% confidence limit | |
| --- | --- | --- | --- |
|  |  | Lower | Upper |
|  | | | |
| **Rheumatoid arthritis diagnosed or treated by doctor in the past 12 months** (N=5897, No N=5817, Yes N=80) | | | |
| Evening-types | 0.9 | 0.3 | 2.4 |
| Intermediate-types | 2.1 | 1.3 | 3.4** |
| **Rheumatic symptoms past month** (N=5903 No N=5473, Yes N=430) | | | |
| Evening-types | 1.4 | 1.0 | 1.9 |
| Intermediate-types | 1.1 | 0.9 | 1.4 |
| **Articular pain** **past month** (N=5898, No N=4060, Yes N=1838) | | | |
| Evening-types | 1.1 | 0.9 | 1.3 |
| Intermediate-types | 1.0 | 0.9 | 1.1 |
| **Other articular disease** **diagnosed or treated by doctor in the past 12 months** (self-reported) (N=5886, No N=5287, Yes N=599) | | | |
| Evening-types | 1.2 | 0.9 | 1.7 |
| Intermediate-types | 0.9 | 0.8 | 1.1 |
| **Medication for articular pain** (N=5805, No N=1534, Yes N=4271) | | | |
| Evening-types | 1.1 | 0.9 | 1.4 |
| Intermediate-types | 1.0 | 0.9 | 1.2 |
| **Spinal disease diagnosed or treated by doctor in the past 12 months** (N=5889, No N=4991, Yes N=898) | | | |
| Evening-types | 1.4 | 1.1 | 1.8** |
| Intermediate-types | 1.0 | 0.9 | 1.2 |
| **Backache past month** (N=5893, No N=3328, Yes N=2565) | | | |
| Evening-types | 1.4 | 1.2 | 1.7*** |
| Intermediate-types | 1.2 | 1.0 | 1.3** |

^a^ Controlled for gender, age, education level, civil status, physical activity, alcohol consumption, current smoking, and depressive symptoms. Morning-types as the reference category. ^*^*p* <0.05; ^**^*p* <0.01; ^***^*p* <0.001; ^****^*p* <0.0001
