# Supplementary material for: Behavioral Trait of Morningness-Eveningness in Association with Articular and Spinal Diseases in a Population
Source: PLoS One. 2014 Dec 3;9(12):e114635. doi: 10.1371/journal.pone.0114635 (PMC4255027; doi:10.1371/journal.pone.0114635)
Supplement: Table S3 — Supplementary analysis including insufficient sleep in the final model. (DOCX) [file pone.0114635.s003.docx]

Table S3. Supplementary analysis including insufficient sleep in the final model.^a^

| Chronotype | Odds ratio | 95% confidence limit | |
| --- | --- | --- | --- |
|  |  | Lower | Upper |
|  | | | |
| **Rheumatoid arthritis diagnosed or treated by doctor in the past 12 months** (N=5939, No N=5858, Yes N=81) | | | |
| Evening-types | 0.8 | 0.3 | 2.1 |
| Intermediate-types | 1.9 | 1.2 | 3.0* |
| **Rheumatic symptoms past month** (N=5946, No N=5512, Yes N=434) | | | |
| Evening-types | 1.3 | 0.9 | 1.8 |
| Intermediate-types | 1.1 | 0.9 | 1.3 |
| **Articular pain** **past month** (N=5941, No N=4083, Yes N=1858) | | | |
| Evening-types | 1.1 | 0.9 | 1.3 |
| Intermediate-types | 1.0 | 0.8 | 1.1 |
| **Other articular disease** **diagnosed or treated by doctor in the past 12 months** (N=5929, No N=5323, Yes N=606) | | | |
| Evening-types | 1.2 | 0.9 | 1.6 |
| Intermediate-types | 0.9 | 0.8 | 1.1 |
| **Medication for articular pain** (N=5843, No N=1541, Yes N=4302) | | | |
| Evening-types | 1.1 | 0.9 | 1.4 |
| Intermediate-types | 1.0 | 0.9 | 1.1 |
| **Spinal disease diagnosed or treated by doctor in the past 12 months** (N=5930, No N=5023, Yes N=907) | | | |
| Evening-types | 1.4 | 1.1 | 1.8* |
| Intermediate-types | 1.0 | 0.9 | 1.2 |
| **Backache past month** (N=5936, No N=3342, Yes N=2594) | | | |
| Evening-types | 1.3 | 1.1 | 1.6** |
| Intermediate-types | 1.1 | 1.0 | 1.3* |

^a^ Controlled for gender, age, education level, civil status, physical activity, alcohol consumption, current smoking, and insufficient sleep. Morning-types as the reference category. ^*^*p* <0.05; ^**^*p* <0.01; ^***^*p* <0.001; ^****^*p* <0.0001
